# Triglyceride-glucose index and subclinical left ventricular dysfunction across cardiovascular-kidney-metabolic syndrome stages: a 7-year retrospective cohort study

**DOI:** 10.3389/fendo.2026.1783741

**Published:** 2026-05-12

**Authors:** Kun-Zhe Tsai, Gen-Min Lin, Chunho Yun, Kuotzu Sung, Charles Jia-Yin Hou, Hung-I Yeh, Nguyen Ngoc Khoi Truong, Chung-Lieh Hung

**Affiliations:** 1 Department of Stomatology of Periodontology, Mackay Memorial Hospital, Taipei, Taiwan; 2 Department of Medicine, MacKay Medical University, New Taipei City, Taiwan; 3 Department of Medicine, Hualien Armed Forces General Hospital, Hualien, Taiwan; 4 Department of Internal Medicine, Tri-Service General Hospital and National Defense Medical University, Taipei, Taiwan; 5 Division of Radiology, MacKay Memorial Hospital, Taipei, Taiwan; 6 Cardiovascular Division, Department of Internal Medicine, Mackay Memorial Hospital, Taipei, Taiwan; 7 International Ph.D. Program in Medicine, College of Medicine, Taipei Medical University, Taipei City, Taiwan; 8 Institute of Biomedical Sciences, MacKay Medical University, New Taipei City, Taiwan

**Keywords:** cardiovascular-kidney-metabolic syndrome, global longitudinal strain, heart failure, subclinical left ventricular dysfunction, triglyceride glucose index

## Abstract

**Background:**

The triglyceride-glucose (TyG) index is associated with cardiovascular disease risk, but its role in identifying subclinical myocardial dysfunction and predicting heart failure (HF) in asymptomatic adults remains unclear.

**Objectives:**

To assess the association between TyG index and left ventricular global longitudinal strain (GLS), define a threshold for subclinical left ventricular dysfunction (SLVD), and evaluate its link to incident HF hospitalization.

**Methods:**

In this retrospective cohort study included, 4,784 asymptomatic adults aged 18–86 years from a Taiwan community and health check-up cohort without lipid-lowering therapy were included. SLVD was defined as GLS ≥–18%. Multivariable logistic and Cox regression analyses examined the relationship between TyG index, SLVD, and HF risk with covariates adjustment.

**Results:**

A TyG index ≥8.64 optimally discriminated individuals with SLVD, with an area under the receiver operating characteristic curve of 0.70. The prevalence of SLVD was 4.9% (130/2,656) among those with a normal TyG index and 12.9% (268/2,067) among those with an elevated TyG index. An elevated TyG index was independently associated with SLVD [odds ratio 1.62; 95% confidence interval (CI): 1.25–2.09], with stronger associations in those with stage B HF or cardiovascular-kidney-metabolic (CKM) syndrome. The proportion of individuals with an elevated TyG index increased markedly across CKM stages, from 3.5% (23/665) at stage 0 to 14.9% (148/991) at stage 1, 62.6% (1,849/2,955) at stage 2, and 42.0% (47/112) at stage 3. Over a median follow-up of 7.43 years, 61 participants experienced HF hospitalization. Individuals with both SLVD and a high TyG index had a substantially higher risk of HF hospitalization (hazard ratio 7.26; 95% CI: 3.44–15.35) compared with those without either condition.

**Conclusions:**

TyG index is a simple, accessible marker for early myocardial dysfunction and future HF risk, particularly when combined with SLVD or early-stage HF.

## Introduction

The recently proposed cardiovascular-kidney-metabolic (CKM) syndrome by the American Heart Association (AHA) underscores the interrelated nature of cardiometabolic and renal dysfunction, with insulin resistance (IR) as a central pathophysiological driver (1, 2). IR contributes to a spectrum of metabolic and vascular abnormalities that accelerate cardiovascular disease progression (1–3). In this context, left ventricular (LV) global longitudinal strain (GLS) has emerged as a sensitive marker of subclinical myocardial dysfunction, enabling detection of myocardial impairment prior to reductions in left ventricular ejection fraction (LVEF) (4).

The triglyceride-glucose (TyG) index, a validated surrogate of IR, has been consistently associated with impaired myocardial deformation, including GLS and other strain indices, independent of traditional risk factors (5–16). Moreover, elevated TyG levels have been linked to incident cardiovascular events, including heart failure (HF), even among individuals without overt cardiovascular disease, suggesting a potential role in early risk stratification (17). However, prior studies have largely focused on populations with established disease or high cardiovascular risk (5–16), and the relationship between the TyG index and subclinical dysfunction across the CKM continuum remains incompletely defined. In addition, clinically relevant thresholds of TyG for identifying GLS defined myocardial impairment have not been established.

Accordingly, we investigated the association between the TyG index and subclinical LV dysfunction (SLVD), as assessed by GLS, in asymptomatic adults without established cardiovascular disease or HF stage C/D (18). We further examined this relationship across CKM stages and sought to derive a clinically relevant TyG threshold for identifying individuals at risk of early myocardial dysfunction, thereby extending risk stratification beyond conventional LVEF-based assessment.

## Methods

### Study population

The study population consisted of 5,715 adults aged 18 to 86 years, derived from two independent cohorts in Taiwan. The first cohort included 1,606 community-based participants from the Mitochondria-Aging in Northern Taiwan (MAGNET) study, which recruited residents from the northern coastal areas of New Taipei City (19). The second cohort comprised 4,109 individuals who underwent Mackay Memorial Hospital cardiovascular health check-up in Taipei City. Medical records from examinations conducted between January 2009 and December 2012 were retrospectively reviewed for anthropometric measurements, hemodynamic parameters, laboratory testing of blood biomarkers, and transthoracic echocardiography. Participants also completed a self-administered questionnaire with regard to substance use, i.e., alcohol consumption, betel-nut chewing, and cigarette smoking, which were classified as active or former/never use. Physical activity within the preceding six months was classified as active or inactive. Medical history, comorbidities, and medication use were obtained from participant’s self-reports and verified against institutional electronic medical records. Approximately 89.6% of participants had accessible records for verification, and a high level of agreement was observed between self-reported conditions and documented diagnoses. Individuals were excluded if they had a history of stage C or stage D HF as defined according to the 2022 AHA/American College of Cardiology (ACC)/Heart Failure Society of America (HFSA) guideline or documented cardiovascular events, were pregnant, or were receiving lipid-lowering therapy (18). The study protocol has been described in detail previously (19, 20).

As a retrospective study design, written informed consent was waived from local institutional ethical review. This study was conducted in accordance with the principles of the Declaration of Helsinki. The study protocol was approved by the Institutional Review Board of Mackay Memorial Hospital (Nos. 14MMHIS172, and 18MMHIS180e).

### Annual health examination

Anthropometric measurements including waist circumference (WC), height, and weight were obtained once with participants standing upright. Body mass index (BMI) was calculated as weight in kilograms divided by height in meters squared (kg/m²). Resting blood pressure was measured in the seated position using an automated oscillometric device. If the initial systolic blood pressure (SBP) or diastolic blood pressure (DBP) was ≥130/80 mmHg, a second measurement was taken after a 15-minute rest. The final value was reported as the average of the two readings.

Morning venous blood samples were collected after an overnight fast of at least 8 hours and analyzed for total cholesterol, low-density lipoprotein cholesterol (LDL-C), high-density lipoprotein cholesterol (HDL-C), triglyceride (TG), fasting plasma glucose (FPG), glycated hemoglobin A1C (HbA1c) and creatinine. Estimated glomerular filtration rate (eGFR) was calculated using the 2007 Modification of Diet in Renal Disease (MDRD) equation (21). TyG index was calculated as *Ln [fasting TG (mg/dL) × FPG (mg/dL)/2]* (22). All laboratory analyses were performed in a central laboratory using standardized protocols with routine quality control procedures. The gold-standard method for assessing insulin resistance is the euglycemic–hyperinsulinemic clamp, which measures peripheral glucose uptake under conditions of elevated insulin concentrations (23). However, this technique is time-consuming, costly, and technically demanding, limiting its use in large epidemiological studies and routine clinical practice. Consequently, simple and reliable surrogate markers of insulin resistance are needed. The TyG index has emerged as a practical surrogate marker for insulin resistance and has been increasingly used in epidemiological studies (7–16, 24–28).

### Transthoracic echocardiographic examination

Transthoracic echocardiography was performed by a single trained sonographer blinded to clinical data, using a commercially available ultrasound system (GE Vivid series, GE Medical Systems, Vingmed, Norway) equipped with a phased-array transducer (2.5–4.5 MHz). Comprehensive cardiac structural and functional assessments were conducted with standard parasternal and apical views obtained in the left lateral decubitus position, following established echocardiographic protocols. Two-dimensional, M-mode, pulsed-wave Doppler, and tissue Doppler imaging (TDI) were acquired from parasternal and apical views. Echocardiographic parameters included heart rate, interventricular septal thickness, LV posterior wall thickness, LV internal dimensions during diastole and systole, peak early (E) and late (A) mitral inflow velocities, E-wave deceleration time, early diastolic mitral annular velocity (e′), and the E/e′ ratio as a surrogate marker of LV filling pressure. TDI-e’ velocity was assessed at the septal mitral annulus from the apical four-chamber view using pulsed-wave tissue Doppler, with the sample volume placed at the junction of the mitral valve annulus and the interventricular septum. LV-GLS was assessed by two-dimensional speckle-tracking echocardiography using LV apical two-, three-, and four-chamber views, and was calculated as the average peak systolic strain (%) across 17 myocardial segments. All echocardiographic measurements were averaged over three consecutive cardiac cycles. In accordance with recent evidence and the latest recommendations from the European Association of Cardiovascular Imaging, SLVD was defined as GLS ≥–18% (3, 7, 29, 30).

The use of a single trained sonographer blinded to clinical data inherently precludes inter-observer variability. LV-GLS was derived using validated semi-automated speckle-tracking software, which further minimizes operator-dependent variability. Although formal intra-observer variability assessment was not performed, the reproducibility of speckle-tracking-derived GLS is well established in guideline-endorsed literature (30), rendering its potential impact on the present findings minimal. Given the observational nature of this study and its reliance on pre-existing data, formal blinding of analysts to TyG index levels was not feasible; nevertheless, analytical integrity was maintained through strict adherence to predefined analytic protocols and the use of objective outcome measures, thereby minimizing the potential for analytical bias.

### Definition of HF

All enrolled participants had preserved ejection fraction and no symptoms or signs of HF. According to the 2022 AHA/ACC/HFSA guideline for the management of HF (18), participants without HF symptoms, structural heart disease, or cardiac biomarkers of stretch or injury (e.g., hypertension, atherosclerotic cardiovascular disease, diabetes, metabolic syndrome and obesity, exposure to cardiotoxic agents, genetic variant for cardiomyopathy, or positive family history of cardiomyopathy) were classified as having stage A HF (at risk for HF). Participants without HF symptoms but with evidence of any of the following: (1) structural heart disease (left atrial volume index ≥29 mL/m², left ventricular mass index > 116 g/m² for men or > 95 g/m² for women, or relative wall thickness > 0.42); (2) increased filling pressures (average E/e′ ≥ 15, septal e′ < 7 cm/sec, lateral e′ <10 cm/sec, or tricuspid regurgitation velocity ≥ 2.8 m/sec); or (3) elevated N-terminal pro-B type natriuretic peptide (NT-proBNP ≥125 pg/mL) were classified as having stage B HF (pre-HF). No individuals with established or refractory HF (stage C or D) were identified.

### Definition of CKM syndrome

In accordance with the 2023 AHA scientific statement on cardiovascular-kidney-metabolic health (1), participants were categorized into CKM stages 0 to 4. Stage 4 included individuals with a self-reported history of heart disease or stroke. Stage 3 encompassed those at very high risk of chronic kidney disease (stage G4 or G5), as defined by the Kidney Disease: Improving Global Outcomes (KDIGO) guidelines (31), including participants with an eGFR <30 mL/min/1.73 m² and/or elevated subclinical heart failure biomarker levels (NT-proBNP ≥125 pg/mL). Stage 2 included individuals with metabolic risk factors, type 2 diabetes mellitus, arterial hypertension (stage 1-2), hypertriglyceridemia (≥135 mg/dL), or reduced kidney function (eGFR <45 mL/min/1.73 m²). Stage 1 were defined by the presence of obesity (BMI ≥23 kg/m²), increased WC (≥80 cm in women or ≥90 cm in men), or prediabetes (glycosylated hemoglobin of 5.7%-6.4% or FPG between 110 and 126 mg/dL). Stage 0 included those with a normal metabolic profile and no history of chronic kidney disease or cardiovascular disease. Individuals classified as stage 4 were excluded from the present analysis.

This study was designed from a preventive medicine perspective and focused on individuals without clinical symptoms of heart failure. The primary aim was to identify high-risk individuals at an early stage and facilitate timely intervention before the development of overt disease. Because CKM stage 4 represents individuals with established cardiovascular disease, including symptomatic HF, these participants were excluded to maintain an asymptomatic cohort and to better evaluate early cardiometabolic risk associated with subclinical cardiac dysfunction.

### Statistical analysis

To assess the potential nonlinear associations between the TyG index and measures of LV structure and function, restricted cubic spline (RCS) regression was constructed with the TyG index treated as a continuous variable. The RCS model with three knots was employed to flexibly model the associations of the TyG index with LV mass index, TDI-e’, E/e’ ratio, and LV-GLS. Overall P values and standardized differences were reported to evaluate the associations and assess potential departures from linearity with age, sex, heart failure, substance use, physical activity, BMI, WC, SBP, DBP, LDL-C, HDL-C, HbA1c, and eGFR. Covariates were selected based on clinical relevance and review of the existing literature.

The diagnostic performance of the TyG index for detecting SLVD was assessed using receiver operating characteristic (ROC) curve analysis, with the area under the curve (AUC) reported. Sensitivity, specificity, and the optimal cutoff value of the TyG index for identifying SLVD, defined as LV-GLS ≥ -18%, were derived from the ROC analysis. The optimal cutoff of the TyG index, 8.64, was derived using Youden’s index from the ROC analysis after full model adjustment. This threshold was subsequently used to stratify participants into low and high TyG index groups (<8.64 and ≥8.64, respectively). Baseline clinical and echocardiographic characteristics were expressed as mean ± standard deviation (SD) for continuous variables and as number (percentage) for categorical variables. Between-group comparisons of continuous variables were performed using one-way analysis of variance (ANOVA), and categorical variables were compared using the chi-square (χ²) test.

To evaluate the association between the TyG index and LV-GLS, multivariable linear regression analysis was conducted with simultaneous adjustment for all covariates. Both the TyG index and LV-GLS were treated as continuous variables. The regression coefficient represents the change in LV-GLS associated with each one-unit increase in the TyG index. To further examine whether the association between the TyG index and LV-GLS differed by HF stages or CKM syndrome, stratified analyses were performed across HF stage A and B, and with or without CKM syndrome. In the HF-stratified models, HF stage was excluded from the covariate adjustment to avoid overadjustment. In addition, multivariable logistic regression was used to estimate the odds ratio (OR) for SLVD associated with a high TyG index, with stratified analyses similarly conducted by HF stage and CKM syndrome.

To evaluate the joint associations of the TyG index and HF stages or CKM syndrome with SLVD, multivariable logistic regression models were constructed with simultaneous adjustment for all covariates. In subgroup analyses stratified by HF stage, HF was excluded as a covariate to avoid overadjustment. For the TyG/HF analysis, participants were categorized into four groups based on TyG index (<8.64 or ≥8.64) and HF stage (stage A or stage B): Group 1, low TyG index and stage A (TyG-/HFa); Group 2, low TyG index and stage B (TyG-/HFb); Group 3, high TyG index and stage A (TyG+/HFa); and Group 4, high TyG index and stage B (TyG+/HFb). Similarly, for the TyG/CKM analysis, participants were grouped according to TyG index and the presence of CKM syndrome: Group 1, low TyG index and without CKM syndrome (TyG−/CKM−); Group 2, low TyG index and with CKM syndrome (TyG−/CKM+); Group 3, high TyG index and without CKM syndrome (TyG+/CKM−); and Group 4, high TyG index and with CKM syndrome (TyG+/CKM+).

Of 3,316 eligible individuals from health check-up cohort (no longitudinal outcomes available in the MAGNET cohort) had follow-up over a median period of 7.43 years (interquartile range: 6.58-8.51 years) to assess the incidence of new-onset HF-related hospitalizations. Multivariable Cox proportional hazards regression models were used to evaluate the associations of combined TyG/SLVD and TyG/HF classifications with the risk of incident HF hospitalization, with adjustment for all covariates. For analyses incorporating the TyG/HF classification, HF stage was excluded from the adjustment model to avoid collinearity. The proportional hazards assumption was assessed by Schoenfeld residual test. Our current Cox models showed no non-proportionality.

Analyses were performed using complete-case data, as the proportion of missing values was low. All statistical analyses were performed using R software (version 4.3.5; R Foundation for Statistical Computing, Vienna, Austria). A two-sided P value < 0.05 was considered statistically significant.

## Results

**Figure 1:**
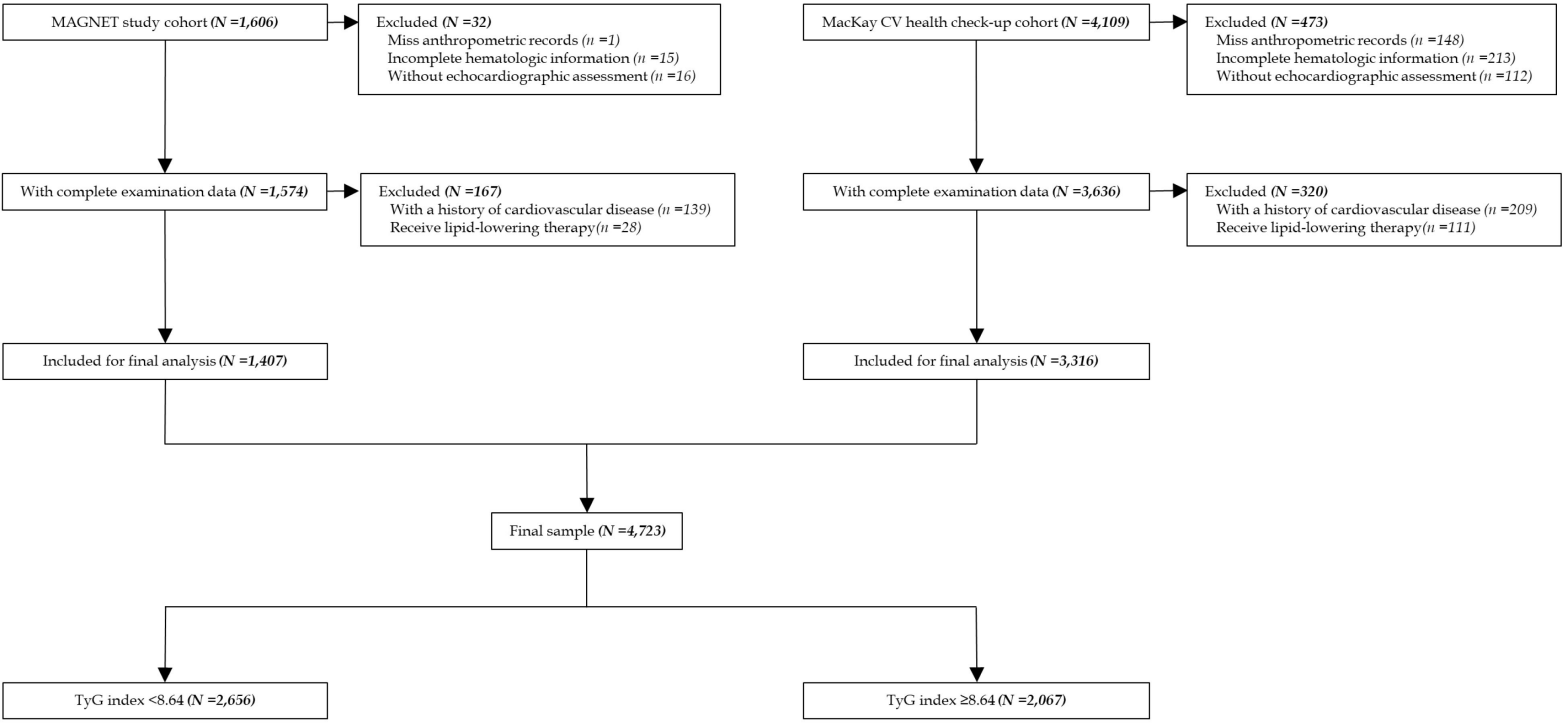
Study flow diagram.

A total of 5,715 participants were initially enrolled, comprising 1,606 from the MAGNET cohort and 4,109 from the health check-up cohort. In the MAGNET cohort, 32 individuals were excluded due to incomplete anthropometric data (n, 1), missing echocardiographic assessments (n, 15), or without echocardiographic assessment (n, 16) leaving 1,574 participants with complete baseline measures. Of these, 167 were further excluded due to a history of CVD (n, 139) or use of lipid-lowering medications (n, 28), resulting in 1,407 eligible participants. In the health check-up cohort, 473 participants were excluded due to missing anthropometric (n, 148), hematologic (n, 213), or echocardiographic (n, 112) records. Among the 3,636 remaining individuals with complete baseline assessments, 320 were further excluded for prevalent CVD (n, 209) or lipid-lowering therapy (n, 111), yielding 3,316 eligible participants. Ultimately, 1,407 participants from MAGNET and 3,316 from health check-up met the inclusion criteria, comprising a final analytic sample of 4,723 individuals (**Figure 1**).

**Supplementary Table 1**. illustrated the ROC curve analysis of TyG index for identifying SLVD, defined as LV-GLS ≥-18%. After adjustment for age, sex, heart failure, substance use, physical activity, BMI, waist circumferences, SBP, DBP, LDL-C, HDL-C, HbA1c, and eGFR, the TyG index yielded an AUC of 0.70 [95% confidence interval (CI), 0.67–0.73] for identifying SLVD. The optimal TyG index cutoff was 8.64, with a sensitivity of 70.4% and a specificity of 59.0%.

**Figure 2:**
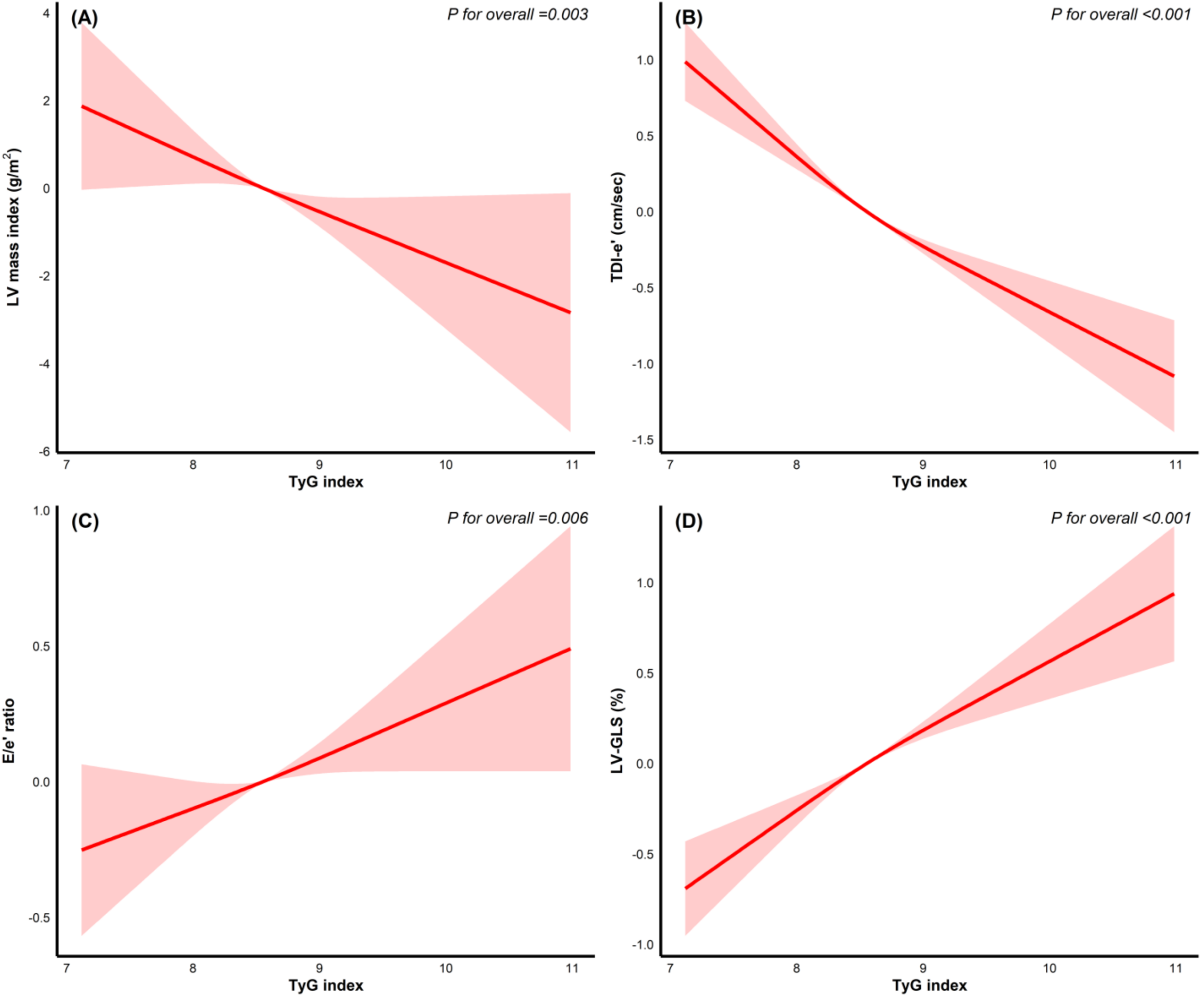
Association between the TyG index and echocardiographic parameters using restricted cubic spline regression. Restricted cubic spline regression curves depicting the association between the TyG index and **(A)** left ventricular (LV) mass index, **(B)** tissue Doppler imaging early diastolic velocity (TDI-e’), **(C)** E/e’ ratio, and **(D)** left ventricular global longitudinal strain (LV-GLS).

**Figure 2** shows the results of RCS regression analyses assessing the association between the TyG index and (A) LV mass index, (B) TDI-e’, (C) E/e’ ratio, and (D) LV-GLS. A significant linear relationship was observed between the TyG index and all four echocardiographic parameters. These linear associations remained evident even in participants without SLVD, as shown in **Supplementary Figures 1A–1D**. However, in those with SLVD, the TyG index was significantly but nonlinear associated only with TDI-e’ and E/e’ ratio (**Supplementary Figures 1F, G**, respectively). Additionally, a consistent linear association was observed between the TyG index and both TDI-e’ and LV-GLS, regardless of whether the TyG index was below or above the threshold of 8.64 (**Supplementary Figure 2**).

**Supplementary Table 2**. displays the associations between each one-unit increase in the TyG index and echocardiographic measures. In the overall population, higher TyG index was significantly associated with lower LV mass index (β =-1.32 and 95% CI -2.06–0.58), lower TDI-e’ [β =-0.54 (-0.64–0.44)], higher E/e’ ratio [β =0.21 (0.08–0.33)], and worse LV-GLS [β =0.38 (0.28–0.48)]. These associations remained generally consistent across HF stages. In HF stage A, the TyG index was significantly associated with all four variables, including LV mass index [β =-1.02 (-1.92–0.12)], TDI-e’ [β =-0.58 (-0.72–0.45)], E/e’ ratio [β, 0.21 (0.07–0.35)], and LV-GLS [β =0.39 (0.26–0.53)]. In HF stage B, significant associations persisted for LV mass index [β =-3.73 (-5.86–1.61)], TDI-e’ [β =-0.46 (-0.69–0.22)], and LV-GLS [β =0.42 (0.17–0.68)], while the association with E/e’ ratio was not significant. When stratified by CKM syndrome, the TyG index was significantly associated with all four variables among those with CKM syndrome, including LV mass index (β =-1.39, -2.31–0.48), TDI-e’ [β =-0.53 (-0.65–0.41)], E/e’ ratio [β, 0.19 (0.03–0.35)], and LV-GLS [β =0.41 (0.28–0.53)]. With regarding to those without CKM syndrome, the relationship persisted for LV mass index [β, -5.49 (-8.62–2.37)], and TDI-e’ [β =-0.47 (-0.94–0.01)], while the association with E/e’ ratio and LV-GLS were not significant. A significant interaction by CKM status was noted only for LV-GLS.

**Table 1** presents the clinical characteristics of the study population stratified by TyG index quartiles. Higher TyG index levels were associated with a progressively greater proportion of men and a higher prevalence of stage B heart failure and advanced CKM syndrome. Participants in the higher TyG quartiles also exhibited a higher prevalence of adverse lifestyle factors, including cigarette smoking, betel-nut chewing, and physical inactivity. In addition, increasing TyG index was associated with an unfavorable cardiometabolic profile, including higher BMI, waist circumference, blood pressure, total cholesterol, LDL-C, triglycerides, fasting glucose, and HbA1c levels, along with lower HDL-C levels. A modest decline in eGFR was also observed across increasing TyG quartiles.

**Table 1:** Clinical characteristics of study population (N, 4,723).

Variables	TyG Quartile 1 <8.14 (n =1,173)	TyG Quartile 2 8.14 – 8.54 (n =1,180)	TyG Quartile 3 8.55 – 8.96 (n =1,177)	TyG Quartile 4 ≥8.97 (n =1,193)	*P*	Standardized differences
TyG index	7.83 ± 0.24	8.35 ± 0.11	8.74 ± 0.12	9.39 ± 0.40	<0.001	4.80
Age, years	48.69 ± 10.40	50.35 ± 10.37	50.19 ± 9.68	50.20 ± 9.37	<0.001	0.16
Sex, %.						
Men	427 (36.4)	652 (55.3)	771 (65.5)	925 (77.5)	<0.001	
Women	746 (63.6)	528 (44.7)	406 (34.5)	268 (22.5)		
Medical history						
Heart failure, %						
Stage A	913 (77.8)	918 (77.8)	884 (75.1)	871 (73.0)	0.01	
Stage B	260 (22.2)	262 (22.2)	293 (24.9)	322 (27.0)		
CKM syndrome, %						
Stage 0	409 (34.9)	193 (16.4)	63 (5.4)	0 (0.0)	<0.001	
Stage 1	344 (29.3)	402 (34.1)	245 (20.8)	0 (0.0)		
Stage 2	390 (33.2)	557 (47.2)	843 (71.6)	1165 (97.7)		
Stage 3	30 (2.6)	28 (2.4)	26 (2.2)	28 (2.3)		
Substance use, %						
Alcohol drinking	368 (31.4)	307 (26.0)	297 (25.2)	313 (26.2)	0.003	
Betel-nut chewing	0 (0.0)	1 (0.1)	3 (0.3)	12 (1.0)	<0.001	
Cigarette smoking	141 (12.0)	155 (13.1)	176 (15.0)	224 (18.8)	<0.001	
Physical activity, %						
Physically inactive	886 (75.5)	946 (80.2)	952 (80.9)	995 (83.4)	<0.001	
Physically active	287 (24.5)	234 (19.8)	225 (19.1)	198 (16.6)		
BMI, kg/m (2)	22.36 ± 2.82	23.80 ± 3.09	25.06 ± 3.35	26.17 ± 3.34	<0.001	1.23
Waist circumferences, cm	76.00 ± 8.29	81.07 ± 8.49	84.42 ± 8.71	88.31 ± 8.54	<0.001	1.46
SBP, mmHg	118.41 ± 16.90	122.72 ± 18.16	125.24 ± 17.45	129.32 ± 17.34	<0.001	0.64
DBP, mmHg	72.58 ± 11.59	75.77 ± 11.65	77.80 ± 11.74	80.57 ± 11.25	<0.001	0.70
Hematologic parameters						
Total cholesterol, mg/dL	194.25 ± 34.68	202.99 ± 34.82	207.35 ± 34.21	215.56 ± 36.63	<0.001	0.60
LDL-C, mg/dL	114.69 ± 29.70	129.80 ± 30.79	137.14 ± 31.13	137.37 ± 35.72	<0.001	0.74
HDL-C, mg/dL	66.67 ± 15.35	58.13 ± 13.77	50.90 ± 11.26	44.44 ± 10.11	<0.001	1.71
Triglyceride, mg/dL	58.11 ± 12.48	90.22 ± 13.33	128.08 ± 20.20	236.44 ± 134.72	<0.001	4.17
Fasting glucose, mg/dL	90.02 ± 8.95	95.68 ± 10.82	100.00 ± 13.12	114.63 ± 37.35	<0.001	0.91
HbA1c, %	5.49 ± 0.36	5.61 ± 0.49	5.72 ± 0.55	6.20 ± 1.29	<0.001	0.75
eGFR, mL/min/1.73m^2^	91.41 ± 19.52	88.41 ± 17.26	87.29 ± 17.89	86.43 ± 17.59	<0.001	0.27

Continuous variables are expressed as mean ± SD (standard deviation), and categorical variables as n (%).

TyG index, triglyceride glucose index; CKM, cardiovascular-kidney-metabolic; BMI, body mass index; SBP, systolic blood pressure; DBP, diastolic blood pressure; LDL-C, low density lipoprotein cholesterol; HDL-C, high density lipoprotein cholesterol; HbA1c, glycated hemoglobin; eGFR, estimated glomerular filtration rate.

**Supplementary Figure 3** depicts the distribution of the proportion of participants with SLVD as well as a high TyG index across CKM stages. A progressive increase in the prevalence of SLVD was observed with advancing CKM stage, rising from 1.8% at stage 0 to 3.9% at stage 1, 11.2% at stage 2, and 13.4% at stage 3. Similarly, the proportion of individuals with a high TyG index increased from 3.5% at stage 0 to 14.9% at stage 1 and peaked at 62.6% at stage 2, before slightly declining to 42.0% at stage 3.

**Table 2** summarizes the echocardiographic characteristics across TyG index quartiles. Higher TyG index levels were associated with progressively higher heart rates, increased LV wall thickness, larger LV internal dimensions, and greater LV mass index, indicating adverse cardiac remodeling. In parallel, indices of diastolic function worsened across TyG quartiles, as reflected by lower tissue Doppler e’ velocities, reduced mitral inflow velocities, and higher E/e’ ratios. LV systolic function showed a subtle decline, with lower ejection fraction and progressively impaired LV-GLS. Accordingly, the proportion of individuals with SLVD increased across TyG quartiles. In contrast, tricuspid regurgitant velocity and NT-proBNP levels showed a decreasing trend across TyG quartiles.

**Table 2:** Echocardiographic characteristics of study population (N, 4,723).

Variables	TyG Quartile 1 <8.14 (n =1,173)	TyG Quartile 2 8.14 – 8.54 (n =1,180)	TyG Quartile 3 8.55 – 8.96 (n =1,177)	TyG Quartile 4 ≥8.97 (n =1,193)	*P*	Standardized differences
Heart rate, beat/min	65.33 ± 10.49	65.51 ± 10.56	67.34 ± 20.00	68.02 ± 11.38	<0.001	0.25
Deceleration time, ms	200.12 ± 36.48	199.80 ± 35.08	201.57 ± 36.58	201.55 ± 34.72	0.49	0.05
Interventricular septum, mm	8.93 ± 10.47	9.03 ± 3.61	9.17 ± 2.37	9.31 ± 1.08	0.40	0.11
LV ejection fraction, %	64.74 ± 5.63	64.25 ± 5.32	63.78 ± 5.60	63.03 ± 5.79	<0.001	0.30
LV posterior wall, mm	8.60 ± 1.06	8.84 ± 1.21	9.05 ± 0.97	9.27 ± 0.95	<0.001	0.67
LV internal dimension in diastole, mm	26.78 ± 20.74	34.11 ± 19.41	35.44 ± 19.17	37.36 ± 18.42	<0.001	0.54
LV internal dimension in systole, mm	16.80 ± 13.05	21.38 ± 12.26	22.23 ± 12.12	23.71 ± 11.97	<0.001	0.55
LV mass index, g/m^2^	74.24 ± 14.68	75.69 ± 14.63	76.41 ± 13.38	77.40 ± 13.80	<0.001	0.22
Relative wall thickness	0.37 ± 0.04	0.38 ± 0.04	0.39 ± 0.04	0.39 ± 0.04	<0.001	0.50
Tissue Doppler imaging e’, cm/sec	10.69 ± 2.46	9.76 ± 2.28	9.32 ± 2.18	8.74 ± 2.06	<0.001	0.86
Mitral E velocity, cm/sec	75.60 ± 16.79	71.09 ± 16.98	68.52 ± 15.18	66.94 ± 14.99	<0.001	0.54
Mitral annulus e’ velocity, cm/sec	10.71 ± 2.48	9.76 ± 2.34	9.34 ± 2.24	8.74 ± 2.15	<0.001	0.85
E/e’ ratio	7.39 ± 2.42	7.62 ± 2.36	7.69 ± 2.38	8.02 ± 2.36	<0.001	0.26
LV-GLS, %						
≤-20%	873 (74.4)	742 (62.9)	627 (53.3)	479 (40.2)	<0.001	
-18.1% - -19.9%	266 (22.7)	363 (30.8)	449 (38.1)	526 (44.1)		
-16.1% - -18%	31 (2.6)	70 (5.9)	90 (7.6)	176 (14.8)		
≥-16%	3 (0.3)	5 (0.4)	11 (0.9)	12 (1.0)		
LA volume index, mL/m^2^	15.36 ± 5.79	15.45 ± 6.21	15.63 ± 6.03	15.85 ± 5.78	0.19	0.08
TR velocity, m/s	2.17 ± 0.34	2.11 ± 0.33	2.07 ± 0.31	2.06 ± 0.32	<0.001	0.33
NT-proBNP, pg/mL	44.54 ± 40.52	41.26 ± 37.45	40.41 ± 139.56	32.45 ± 33.45	0.02	0.33

Continuous variables are expressed as mean ± SD (standard deviation), and categorical variables as n (%).

TyG index, triglyceride glucose index; LV, left ventricle; LV-GLS, left ventricle function global longitudinal strain; LA, left atrial; TR, tricuspid regurgitant; NT-proBNP, N-terminal pro-B type natriuretic peptide.

**Table 3** revealed the associations between a high TyG index and SLVD. In the overall population, individuals with a high TyG index demonstrated significantly greater odds of SLVD (OR, 1.62 and 95% CI: 1.25–2.09). When stratified by HF stages, the association remained significant in participants with stage A HF [OR, 2.02 (1.40–2.91)], but not in those with stage B HF. Regarding CKM classification, a significant association was observed in participants with CKM syndrome [OR, 1.67 (1.24–2.24)], whereas no such relationship was found in those without it. Notably, when further stratified by CKM stage, a high TyG index was associated with increased odds of SLVD only in individuals with stage 2 CKM syndrome [OR, 1.45 (1.07–1.95)]. There were no significant interactions between high TyG index and either HF stages (*P* for interaction, 0.75) or CKM syndrome (*P* for interaction, 0.42).

**Table 3:** Stratification analysis on the association of high TyG index with subclinical left ventricular dysfunction, defined as LV-GLS ≥–18%.

Variables	N	SLVD	OR (95% CI)	*P*	*P* for interaction
Total	4,723	398	1.62 (1.25 – 2.09)	<0.001	
Heart failure					
Stage A	3,586	262	2.02 (1.40 – 2.91)	<0.001	0.75
Stage B	1,137	136	1.31 (0.79 – 2.15)	0.29	
CKM syndrome					
Without	665	12	4.40 (0.24 – 79.83)	0.31	0.42
With	4,058	386	1.67 (1.24 – 2.24)	0.001	
Stage 0	665	12	6.51 (0.91 – 46.83)	0.063	Ref.
Stage 1	991	39	1.48 (0.66 – 3.34)	0.34	0.33
Stage 2	2,955	332	1.45 (1.07 – 1.95)	0.01	0.31
Stage 3	112	15	1.30 (0.24 – 7.06)	0.75	0.27

Data are presented as odds ratios (OR) and 95% confidence intervals (CI) using multiple logistic regression analysis with age, sex, heart failure, substance use, physical activity, BMI, waist circumferences, SBP, DBP, LDL-C, HDL-C, HbA1c, and eGFR adjustment.

CKM, cardiovascular-kidney-metabolic; SLVD, subclinical left ventricular dysfunction.

**Table 4** shows the associations of combined TyG/HF and TyG/CKM syndrome status with the odds of SLVD. Compared with the TyG−/HFa group, there was a stepwise increase in the odds of SLVD across the TyG−/HFb, TyG+/HFa, and TyG+/HFb groups in the univariate model [OR, 1.98 (1.29–3.03), 3.60 (2.64–4.90), and 5.43 (3.81–7.75), respectively; *P* for trend <0.001]. After multivariable adjustment, the TyG+/HFb group remained significantly associated with the highest odds of SLVD [OR, 2.19 (1.46–3.27)], followed by the TyG+/HFa group [OR, 1.78 (1.25–2.52)]. The association in the TyG−/HFb group was attenuated and no longer statistically significant [OR, 1.34 (0.86–2.10)], although the overall trend remained significant (*P* for trend < 0.001).

**Table 4:** Association between TyG index and subclinical left ventricular dysfunction with and without coexisting stage B heart failure or CKM syndrome.

Variables	N	SLVD	Univariable model		Multivariable model	
			OR (95% CI)	*P*	OR (95% CI)	*P*
Heart failure						
Low TyG and stage A HF (TyG-/HFa)	2,056	85	1.00		1.00	
Low TyG and stage B HF (TyG-/HFb)	600	45	1.98 (1.29 – 3.03)	0.002	1.34 (0.86 – 2.10)	0.20
High TyG and stage A HF (TyG+/HFa)	1,530	177	3.60 (2.64 – 4.90)	<0.001	1.78 (1.25 – 2.52)	0.001
High TyG and stage B HF (TyG+/HFb)	537	91	5.43 (3.81 – 7.75)	<0.001	2.19 (1.46 – 3.27)	<0.001
* P* for trend				<0.001		<0.001
CKM syndrome						
Neither high TyG nor CKM syndrome (TyG-/CKM-)	642	10	1.00		1.00	
CKM syndrome only (TyG-/CKM+)	2,014	120	3.97 (2.07 – 7.62)	<0.001	1.33 (0.67 – 2.64)	0.41
High TyG index only (TyG+/CKM-)	23	2	6.02 (1.24 – 29.20)	0.02	4.50 (0.91 – 22.20)	0.065
Both high TyG and CKM syndrome (TyG+/CKM+)	2,044	266	9.47 (5.01 – 17.92)	<0.001	2.10 (1.04 – 4.21)	0.03
* P* for trend				<0.001		<0.001

Data are presented as odds ratios (OR) and 95% confidence intervals (CI) using multiple logistic regression analysis with age, sex, heart failure, substance use, physical activity, BMI, waist circumferences, SBP, DBP, LDL-C, HDL-C, HbA1c, and eGFR adjustment.

HF, heart failure; TyG-, TyG index <8.64; TyG+, TyG index ≥8.64; HFa, Stage A heart failure; HFb, Stage B heart failure; CKM, cardiovascular-kidney-metabolic; CKM-, without CKM syndrome; CKM+, with CKM syndrome; SLVD, subclinical left ventricular dysfunction.

With respect to the TyG/CKM classification, the univariate analysis also demonstrated progressively higher odds of SLVD in the TyG−/CKM+, TyG+/CKM−, and TyG+/CKM+ groups [OR, 3.97 (2.07–7.62), 6.02 (1.24–29.20), and 9.47 (5.01–17.92), respectively; *P* for trend < 0.001], using the TyG−/CKM− group as the reference. However, in the fully adjusted model, only the TyG+/CKM+ group remained significantly associated with increased odds of SLVD [OR, 2.10 (1.04–4.21)].

**Figure 3:**
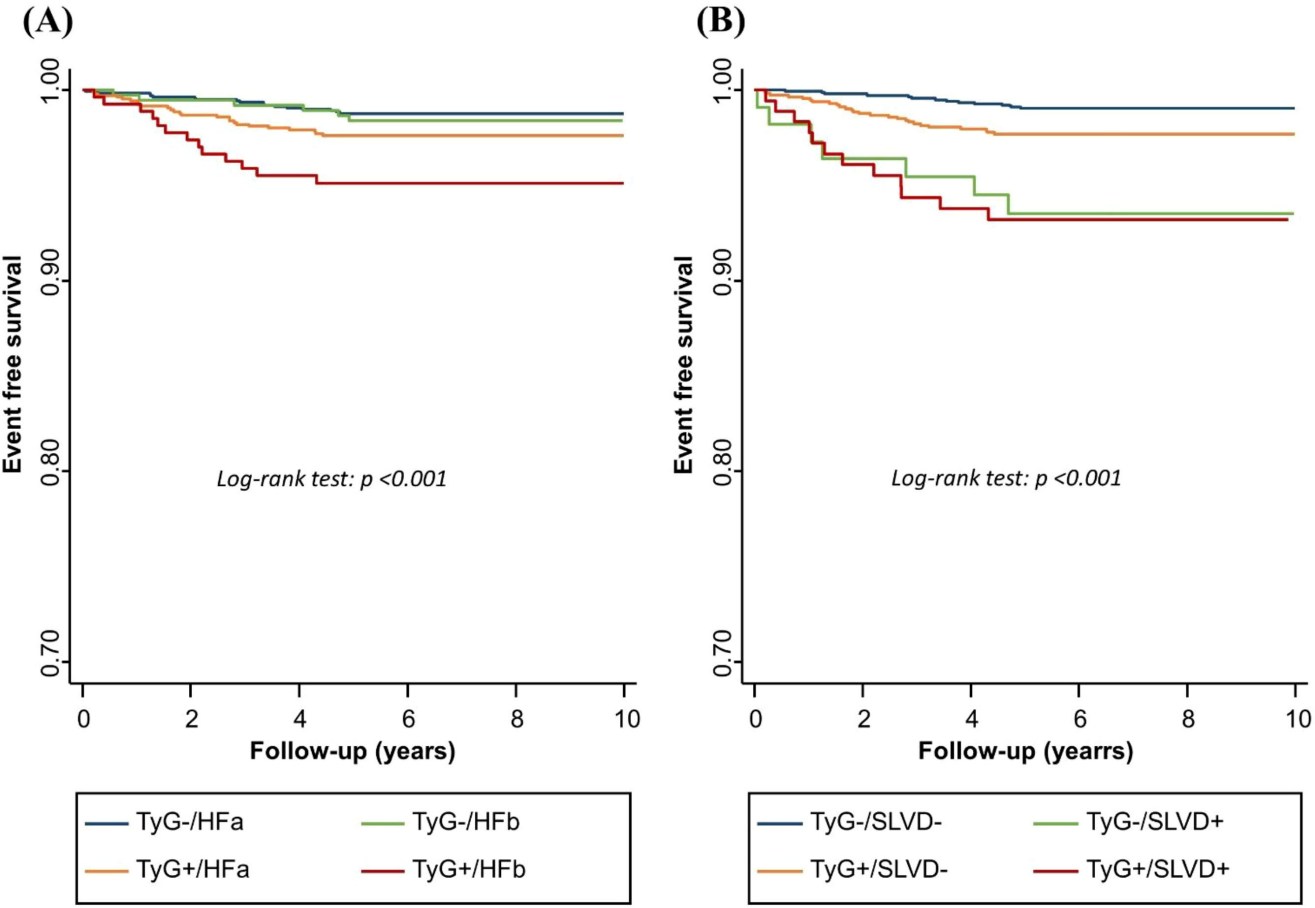
Kaplan–Meier analysis of heart failure–related hospitalization stratified by **(A)** baseline TyG index and heart failure stage, and **(B)** baseline TyG index and subclinical left ventricular dysfunction.

**Table 5** reports adjusted hazard ratios from multivariate Cox models, while **Figure 3** displays the corresponding Kaplan–Meier curves for HF-related hospitalization based on combined TyG/HF and TyG/SLVD classifications. A total of 61 participants were hospitalized for HF over a median follow-up of 7.43 years. Individuals with both a high TyG index and stage B HF demonstrated the highest cumulative incidence of hospitalization for HF (**Figure 3A**) [HR: 1.50 (0.62-3.63), 1.96 (1.06-3.62), 3.78 (1.81-7.92) for TyG-/HFb, TyG+/HFa, and TyG+/HFb respectively; TyG-/HFa as reference]. Similarly, those with both a high TyG index and SLVD experienced the greatest risk of HF-related hospitalization (**Figure 3B**) [HR: 6.93 (2.85-16.85), 2.39 (1.28-4.45), 7.26 (3.44-15.35) for TyG-/SLVD+, TyG+/SLVD-, and TyG+/SLVD+ respectively; TyG-/SLVD- as reference].

**Table 5:** Association of abnormality of TyG index, subclinical left ventricular dysfunction, and heart failure stage with risk of new onset heart failure related hospitalization.

Variables	N	Event	HR (95% CI)	*P*
HF Stage				
Low TyG and stage A HF (TyG-/HFa)	1,489	17	1.00	
Low TyG and stage B HF (TyG-/HFb)	415	7	1.50 (0.62 – 3.63)	0.36
High TyG and stage A HF (TyG+/HFa)	1,128	25	1.96 (1.06 – 3.62)	0.03
High TyG and stage B HF (TyG+/HFb)	284	12	3.78 (1.81 – 7.92)	<0.001
* P* for trend				<0.001
SLVD				
Neither high TyG nor SLVD (TyG-/SLVD-)	1,787	16	1.00	
SLVD only (TyG-/SLVD+)	117	7	6.93 (2.85 – 16.85)	<0.001
High TyG index only (TyG+/SLVD-)	1,221	26	2.39 (1.28 – 4.45)	0.006
Both high TyG and SLVD (TyG+/SLVD+)	191	12	7.26 (3.44 – 15.35)	<0.001
* P* for trend				<0.001

Data are presented as hazard ratios (HR) and 95% confidence intervals (CI) using multiple logistic regression analysis with age, sex, heart failure, substance use, physical activity, BMI, waist circumferences, SBP, DBP, LDL-C, HDL-C, HbA1c, and eGFR adjustment.

SLVD, subclinical left ventricular dysfunction; SLVD-, without subclinical left ventricular dysfunction; SLVD+, with subclinical left ventricular dysfunction; TyG-, TyG index <8.64; TyG+, TyG index ≥8.64; HF, heart failure; HFa, stage A heart failure; HFb, stage B heart failure.

## Discussion

In this community-based cohort of 4,784 asymptomatic adults without established CVD and not receiving lipid-lowering therapy, the TyG index was linearly associated with worsening myocardial function, with each one-unit increase corresponding to a 0.38% reduction in LV-GLS. Although modest in absolute terms, even small differences in GLS are clinically meaningful, as they reflect early myocardial dysfunction and have been linked to future heart failure risk. These findings suggest that incremental increases in insulin resistance may translate into measurable impairment in myocardial mechanics at a subclinical stage. The adverse impact became more pronounced above the threshold of 8.64, beyond which the odds of SLVD (GLS ≥-18%) increased by 60%. While stage B HF alone did not significantly elevate this HF risk, its combination with a high TyG index (≥8.64) conferred a markedly greater HF risk. SLVD was also independently associated with HF-related hospitalization. Individuals with both a high TyG index and SLVD had a significantly increased risk of future HF hospitalization, more than seven-fold higher, compared to those with neither abnormality, underscoring the synergistic contribution of IR and early myocardial impairment in HF pathogenesis. (**Supplementary Figure 4**) Although the discriminative ability of the TyG index was modest (AUC, 0.70), such performance is consistent with many risk markers used for population-level cardiovascular risk stratification (7, 12, 15, 17). In this context, the clinical relevance of the TyG index may lie less in diagnostic accuracy and more in its ability to identify individuals at higher likelihood of subclinical myocardial dysfunction before the onset of overt HF.

Current evidence linking the TyG index to cardiovascular pathology is largely derived from large-scale prospective studies focused on predicting overt CVD events. These studies consistently demonstrate that the TyG index is a robust predictor of incident CVD and cardiovascular mortality among individuals without pre-existing CVD (24–27). Moreover, TyG-derived indices that incorporate anthropometric measures, such as TyG-BMI and TyG-WC, have shown superior prognostic performance compared with the TyG index alone (24–26). In addition, another population-based cohorts has reported a U-shaped relationship between the TyG index and CVD mortality, suggesting elevated risk at both low and high ends of the index distribution (27). These findings underscore the clinical utility of the TyG index in identifying individuals at risk for overt cardiovascular events. Our study further found a significant association between the TyG index and SLVD, a preclinical manifestation of myocardial impairment. In particular, individuals with both a high TyG index and stage B HF exhibited a markedly greater likelihood of SLVD. This synergistic effect may reflect the additive burden of cardiac remodeling and IR may accelerate myocardial injury.

Given the simplicity and accessibility of calculating the TyG index, its application may be particularly feasible in healthcare settings with limited resources and among populations with relatively disadvantaged socioeconomic backgrounds. Notably, a mega prospective cohort of 3.5 million participants in the Chinese mainland indicated that the TyG index cut-off values were 9.75 for all-cause death and 9.85 for cardiovascular death (28), which is substantially higher than the threshold of 8.64 observed in our study for detecting SLVD. While part of this difference may stem from the variation in clinical endpoints, it may also reflect the unique cardiometabolic vulnerability of our study population. In Taiwan, many working adults come from economically modest backgrounds and are exposed in adulthood to increasingly modern, urbanized environments characterized by sedentary lifestyles, socioeconomic inequality, and diets rich in caloric density. These rapid environmental transitions may contribute to a mismatch between earlier life conditions and adult exposures, ultimately heightening susceptibility to insulin resistance and cardiovascular dysfunction. Our findings suggest that among general ethnic Asian populations, a lower TyG index threshold may be clinically appropriate for early identification of subclinical cardiac dysfunction and for optimizing primary prevention strategies. Based on these findings, we recommend the TyG index as a simple, practical, and clinically meaningful screening tool for the early detection of subclinical myocardial impairment. Further investigations are still necessary to thoroughly understand the prognostic implications of the TyG index for subclinical myocardial impairment.

In the present study, we extend prior evidence linking the TyG index to subclinical myocardial dysfunction by demonstrating a consistent association with impaired LV-GLS in asymptomatic individuals without established cardiovascular disease. Prior studies, largely cross-sectional and conducted in China, have also reported positive associations between the worsed TyG index and impaired LV-GLS, but have predominantly focused on high-risk populations, including those with diabetes, obesity, or chronic HF, thereby limiting generalizability to broader, lower-risk populations (7–16). Our findings address this gap by showing that the association between TyG and SLVD is evident even among asymptomatic adults without established cardiovascular disease or lipid-lowering therapy, suggesting that metabolic dysregulation may contribute to myocardial impairment early in the disease continuum. Notably, even within the broader construct of IR, differential associations have been reported. In a community-based population with preserved left ventricular ejection fraction (≥50%), elevated HOMA-IR demonstrated a robust association with subclinical left ventricular systolic dysfunction, whereas the TyG index exhibited a more pronounced correlation with early diastolic dysfunction (13). Taken together, these findings, along with our results, support the concept that the TyG index may serve as a practical, population-level marker of early myocardial impairment, extending its relevance beyond traditionally defined high-risk groups.

IR, as reflected by an elevated TyG index, contributes to subclinical myocardial dysfunction through interconnected metabolic, inflammatory, and neurohormonal pathways. Excess free fatty acids and lipotoxicity impair mitochondrial energetics, while chronic inflammation and oxidative stress promote endothelial and microvascular dysfunction (32–37). Concurrent activation of the renin–angiotensin–aldosterone system (RAAS) further induces myocardial fibrosis and adverse remodeling (38). These processes preferentially impair longitudinal myocardial function, resulting in early abnormalities detectable by reduced GLS despite preserved left ventricular ejection fraction (4). Along the CKM continuum, increasing metabolic burden and progression from HF stage A to B amplify these effects, facilitating the transition from subclinical dysfunction to overt HF. Although CKM syndrome alone was not independently associated with increased odds of SLVD, the coexistence of a high TyG index and CKM syndrome conferred a synergistic effect, further elevating the likelihood of myocardial dysfunction. CKM syndrome typically commences with the interaction of impaired glucose regulation, excessive or dysfunctional obesity (1). Emerging studies suggest that diabetic cardiomyopathy may begin with a latent phase characterized by subtle myocardial dysfunction in the absence of overt clinical symptoms (7, 39). Epidemiologic estimates indicate that 50% to 67% of individuals with diabetes may exhibit SLVD (40, 41), with strain imaging detecting subtle systolic impairment in approximately one-third of these individuals (41). Interestingly, while a diagnosis of diabetes per se has not been uniformly associated with SLVD, accumulating evidence indicates a stepwise deterioration in GLS with increasing glycemic burden, particularly with rising hemoglobin A1c levels (42–44). These findings highlight the potential of chronic hyperglycemia, rather than the binary presence of diabetes, as a more accurate determinant of early myocardial contractile impairment.

This study benefits from a large sample size, standardized data collection by specially trained personnel, and structured documentation of clinical outcomes. Myocardial deformation was assessed in a dedicated study center, and the reproducibility of LV-GLS measurements was confirmed to be acceptable. However, some limitations should be acknowledged when interpreting the findings of this study. First, participants were drawn from a health check-up cohort and were not randomly selected, which may introduce selection bias and limit generalizability; however, the relatively large sample with a broad spectrum of cardiometabolic risk profiles may partially mitigate this concern. Second, the single-center, cross-sectional design precludes causal inference between the TyG index and SLVD. Third, despite comprehensive covariate adjustment, residual confounding from unmeasured factors cannot be excluded. Fourth, although speckle-tracking echocardiography is widely used, its accuracy may be affected by suboptimal image quality in certain individuals. Fifth, kidney function was defined solely by eGFR without incorporating albuminuria, which may result in misclassification of CKM stages. Finally, reliance on self-reported data introduces potential recall bias.

## Conclusion

A higher TyG index was independently associated with impaired LV-GLS and an increased risk of incident HF-related hospitalization in asymptomatic adults without existing CVD or lipid-lowering therapy. A TyG index threshold of ≥8.64 offered optimal discrimination for identifying SLVD. The adverse myocardial effects of IR, as reflected by an elevated TyG index, were further accentuated in the presence of stage B HF or CKM syndrome. Individuals with both SLVD and a high TyG index had an approximately seven-fold higher risk of future HF events compared with those without either condition. These findings support the TyG index as a simple, accessible marker for early cardiovascular risk stratification. Early intervention targeting IR may help preserve myocardial function and delay progression to overt HF.

## Data Availability

The original contributions presented in the study are included in the article/**Supplementary Material**. Further inquiries can be directed to the corresponding author.

## Supplementary material

Supplementary Figure 1 Association Between the TyG Index and Echocardiographic Parameters in Participants With and Without Subclinical Left Ventricular Dysfunction Linear associations persisted in participants without subclinical left ventricular dysfunction (SLVD), while nonlinear associations with TDI-e’ and E/e’ ratio were observed among those with SLVD

Supplementary Figure 2 Association Between the TyG Index and Echocardiographic Parameters Stratified by TyG Threshold Restricted cubic spline regression showing consistent linear associations between the TyG index and LV mass index, TDI-e’, E/e’ ratio, and LV-GLS, stratified by TyG index <8.64 **(A–D)** and ≥8.64 **(E–H)**.

Supplementary Figure 3 Distribution of Subclinical Left Ventricular Dysfunction and High TyG Index Across CKM Stage 0-3

Supplementary Figure 4 Association of IR, Subclinical Myocardial Dysfunction, and HF Hospitalization. Elevated IR, as indicated by a TyG index ≥8.64, is associated with the risk of SLVD, defined by LV-GLS ≥–18%. SLVD serves as an intermediate phenotype linking metabolic dysregulation to subsequent HF hospitalization. The progression from IR to HF is further modified and amplified by CKM syndrome and advancement from stage A to stage B HF, demonstrating a synergistic effect that increases susceptibility to adverse clinical outcomes.

Supplementary Table 1 Receiver Operating Characteristic Curve Analysis and Optimal Cutoff of TyG Index in Identifying Subclinical Left Ventricular Dysfunction.With age, sex, heart failure, substance use, physical activity, BMI, waist circumferences, SBP, DBP, LDL-C, HDL-C, HbA1c, and eGFR adjustment.

Supplementary Table 2 The Associations of Each Unit Increase in the TyG Index With Cardiac Variables.Data are presented as β value and 95% confidence intervals (CI) using multiple linear regression analysis with age, sex, heart failure, substance use, physical activity, BMI, waist circumferences, SBP, DBP, LDL-C, HDL-C, HbA1c, and eGFR adjustment. HF, heart failure; CKM, cardiovascular-kidney-metabolic; LV, left ventricular; TDI, tissue Doppler image; LV-GLS, left ventricle function global longitudinal strain.

## Glossary

CKM: cardiovascular-kidney-metabolic
AHA: American Heart Association
IR: insulin resistance
LV: left ventricular
GLS: global longitudinal strain
LVEF: left ventricular ejection fraction
TyG: triglyceride-glucose
CVD: cardiovascular disease
SLVD: subclinical left ventricular dysfunction
HF: heart failure
MAGNET: Mitochondria-Aging in Northern Taiwan
ACC: American College of Cardiology
HFSA: Heart Failure Society of America
WC: waist circumferences
BMI: body mass index
SBP: systolic blood pressure
DBP: diastolic blood pressure
LDL-C: low-density lipoprotein cholesterol
HDL-C: high-density lipoprotein cholesterol
TG: triglyceride
FPG: fasting plasma glucose
HbA1c: glycated hemoglobin
eGFR: estimated glomerular filtration rate
MDRD: Modification of Diet in Renal Disease
TDI: tissue Doppler imaging
NT-proBNP: N-terminal pro-B type natriuretic peptide
KDIGO: Kidney Disease: Improving Global Outcomes
RCS: restricted cubic spline
ROC: receiver operating characteristic
AUC: area under the curve
SD: standard deviation
ANOVA: analysis of variance
χ²: chi-square
OR: odds ratio
CI: confidence interval
TyG-: low triglyceride-glucose index
TyG+: high triglyceride-glucose index
HFa: stage A heart failure
HFb: stage B heart failure
CKM-: without cardiovascular-kidney-metabolic syndrome
CKM+: with cardiovascular-kidney-metabolic syndrome
SLVD-: without subclinical left ventricular dysfunction
SLVD+: with subclinical left ventricular dysfunction
RAAS: renin–angiotensin–aldosterone system.
