# Correction: Triglyceride-glucose index and subclinical left ventricular dysfunction across cardiovascular-kidney-metabolic syndrome stages: a 7-year retrospective cohort study

**DOI:** 10.3389/fendo.2026.1914586

**Published:** 2026-07-13

**Authors:** Kun-Zhe Tsai, Gen-Min Lin, Chunho Yun, Kuotzu Sung, Charles Jia-Yin Hou, Hung-I Yeh, Nguyen Ngoc Khoi Truong, Chung-Lieh Hung

**Affiliations:** 1 Department of Stomatology of Periodontology, Mackay Memorial Hospital, Taipei, Taiwan; 2 Department of Medicine, MacKay Medical University, New Taipei City, Taiwan; 3 Department of Medicine, Hualien Armed Forces General Hospital, Hualien, Taiwan; 4 Department of Internal Medicine, Tri-Service General Hospital and National Defense Medical University, Taipei, Taiwan; 5 Division of Radiology, MacKay Memorial Hospital, Taipei, Taiwan; 6 Cardiovascular Division, Department of Internal Medicine, Mackay Memorial Hospital, Taipei, Taiwan; 7 International Ph.D. Program in Medicine, College of Medicine, Taipei Medical University, Taipei City, Taiwan; 8 Institute of Biomedical Sciences, MacKay Medical University, New Taipei City, Taiwan

**Keywords:** cardiovascular-kidney-metabolic syndrome, global longitudinal strain, heart failure, subclinical left ventricular dysfunction, triglyceride glucose index

The ethics approval number was erroneously given as “No. 17MMHIS082e”. The correct approval number is “Nos. 14MMHIS172, and 18MMHIS180e”.

The original version of this article has been updated.

