# Supplementary material for: Triglyceride-glucose index and subclinical left ventricular dysfunction across cardiovascular-kidney-metabolic syndrome stages: a 7-year retrospective cohort study
Source: Front Endocrinol (Lausanne). 2026 May 12;17:1783741. doi: 10.3389/fendo.2026.1783741 (PMC13201156; doi:10.3389/fendo.2026.1783741)
Supplement: Supplementary Figure 1 — Association Between the TyG Index and Echocardiographic Parameters in Participants With and Without Subclinical Left Ventricular Dysfunction Linear associations persisted in participants without subclinical left ventricular dysfunction (SLVD), while nonlinear associations with TDI-e’ and E/e’ ratio were observed among those with SLVD [file DataSheet1.docx]

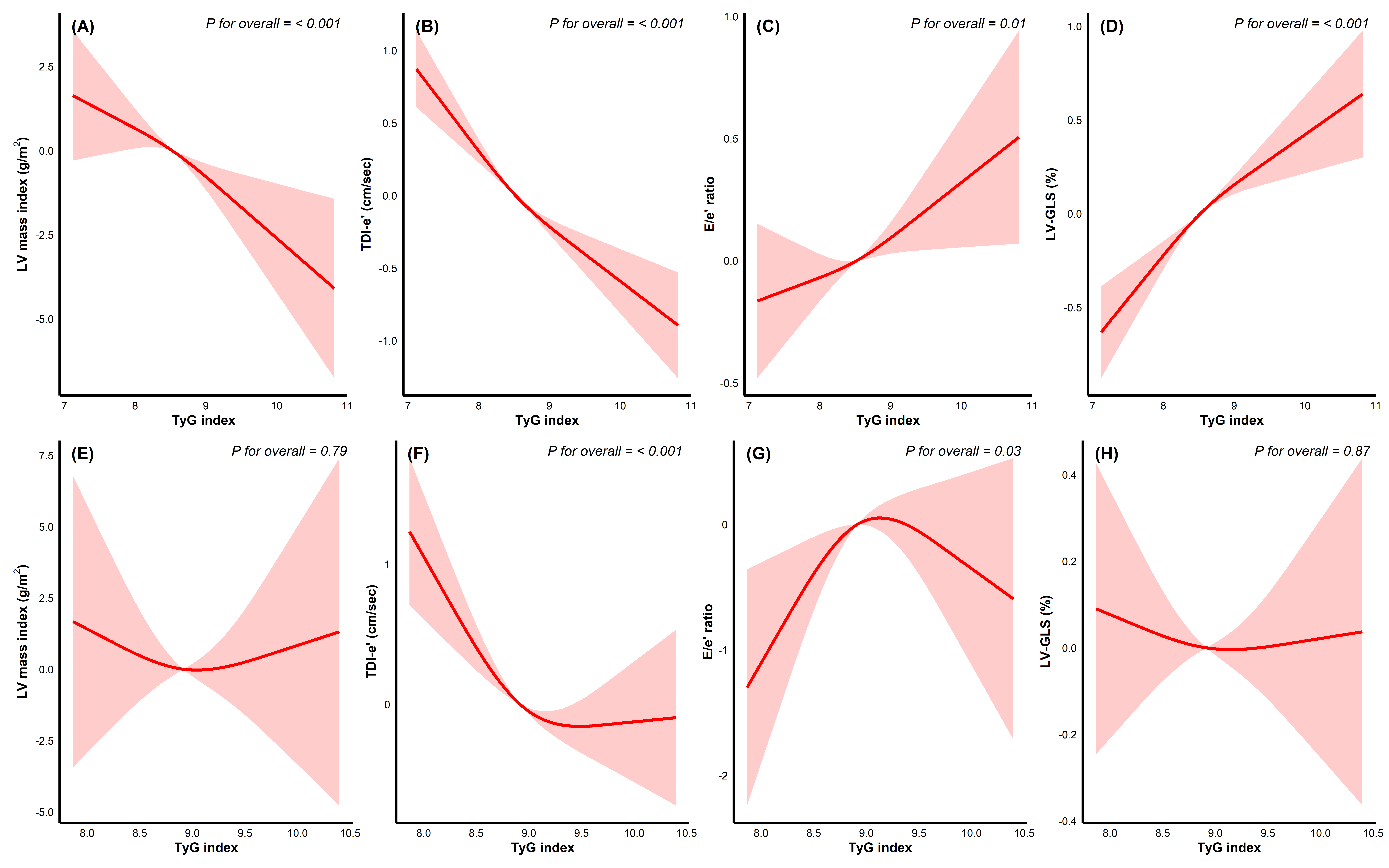


**SUPPLEMENTAL FIGURE 1. Association Between the TyG Index and Echocardiographic Parameters in Participants With and Without Subclinical Left Ventricular Dysfunction**

Linear associations persisted in participants without subclinical left ventricular dysfunction (SLVD), while nonlinear associations with TDI-e’ and E/e’ ratio were observed among those with SLVD


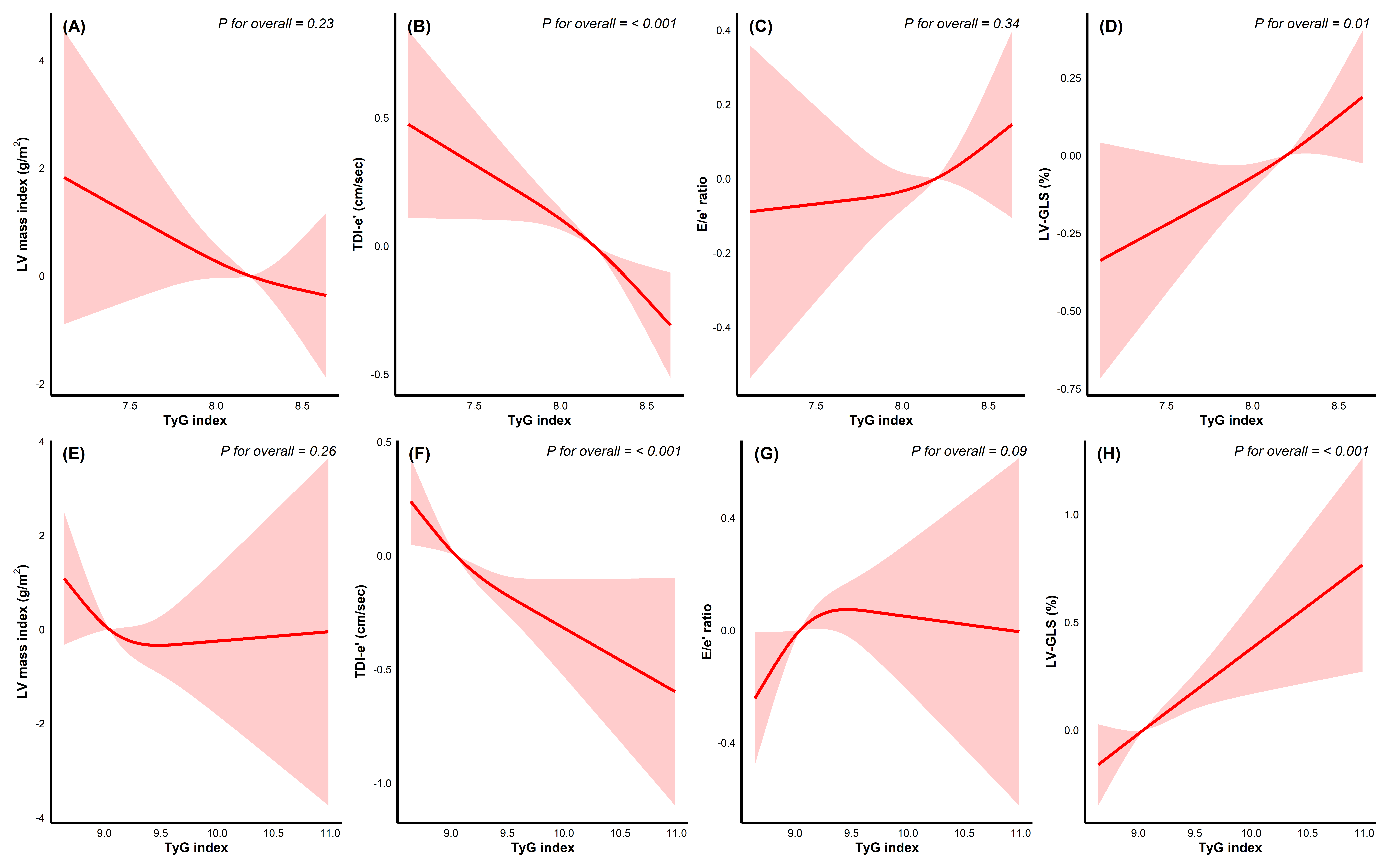


**SUPPLEMENTAL FIGURE 2. Association Between the TyG Index and Echocardiographic Parameters Stratified by TyG Threshold**

Restricted cubic spline regression showing consistent linear associations between the TyG index and LV mass index, TDI-e’, E/e’ ratio, and LV-GLS, stratified by TyG index <8.64 (A–D) and ≥8.64 (E–H).


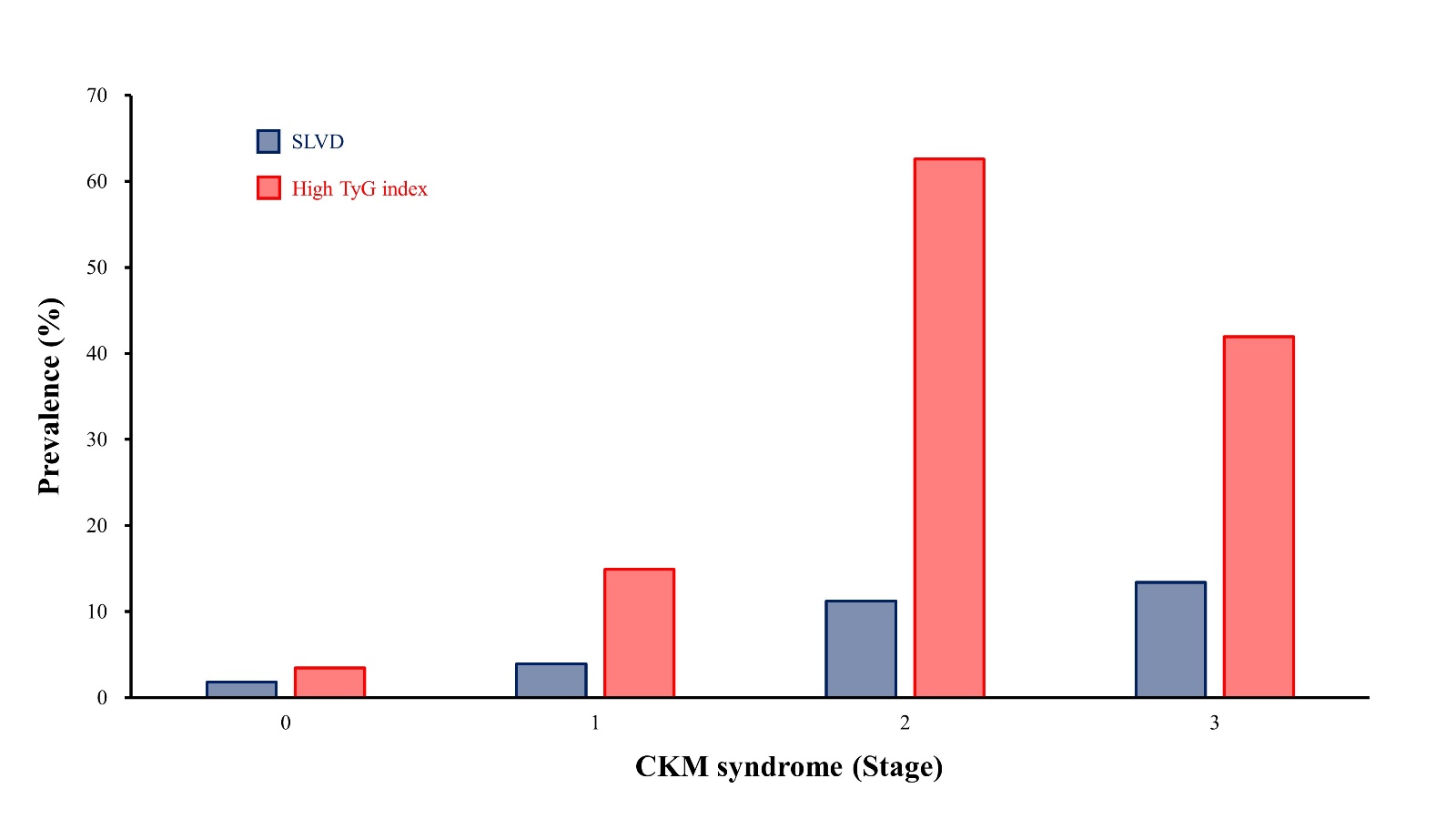


**SUPPLEMENTAL FIGURE 3. Distribution of Subclinical Left Ventricular Dysfunction and High TyG Index Across CKM Stage 0-3**


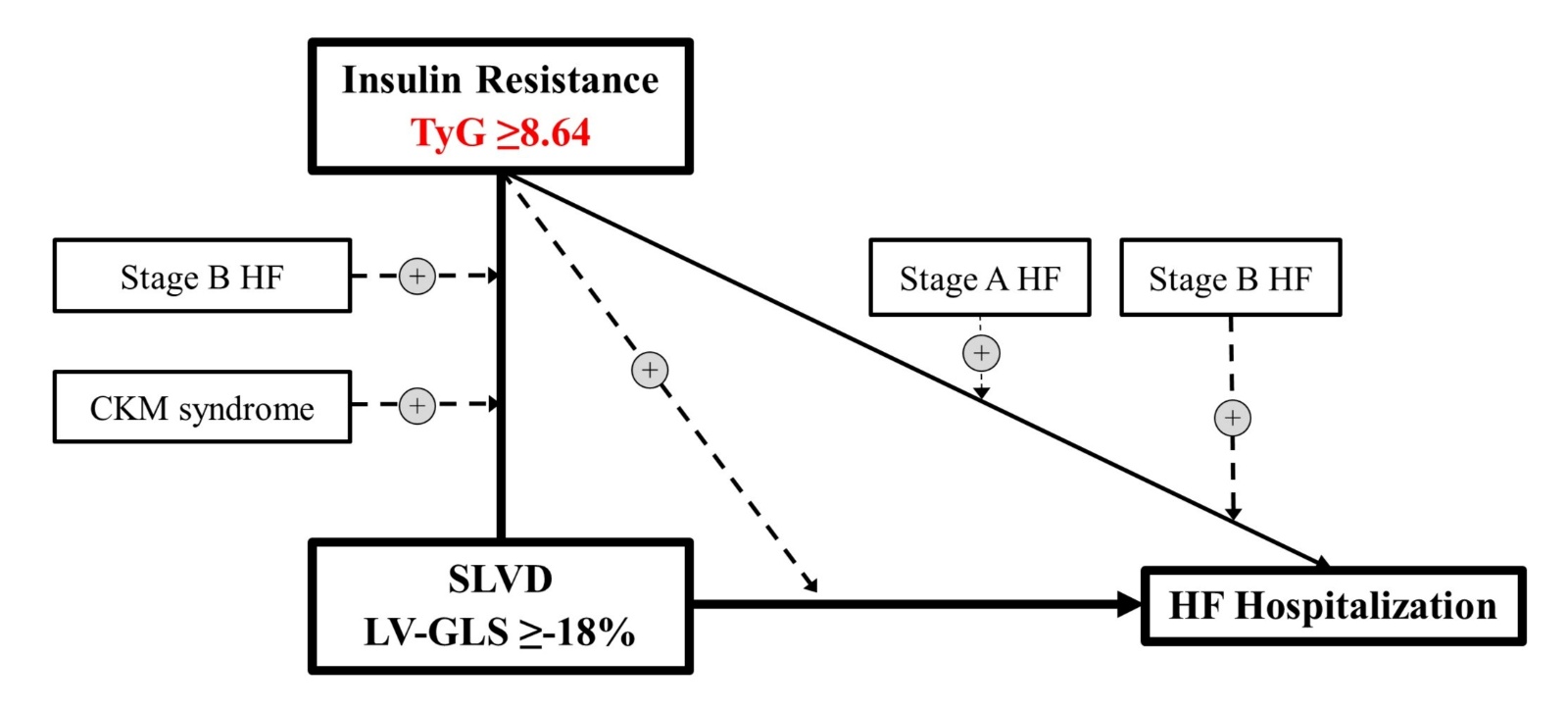


**SUPPLEMENTAL FIGURE 4. Association of IR, Subclinical Myocardial Dysfunction, and HF Hospitalization.**

Elevated IR, as indicated by a TyG index ≥8.64, is associated with the risk of SLVD, defined by LV-GLS ≥–18%. SLVD serves as an intermediate phenotype linking metabolic dysregulation to subsequent HF hospitalization. The progression from IR to HF is further modified and amplified by CKM syndrome and advancement from stage A to stage B HF, demonstrating a synergistic effect that increases susceptibility to adverse clinical outcomes.

**SUPPLEMENTAL TABLE 1. Receiver Operating Characteristic Curve Analysis and Optimal Cutoff of TyG Index in Identifying Subclinical Left Ventricular Dysfunction.**

|  | Cutoff point | Sensitivity | Specificity | AUC | 95% CI | *P* |
| --- | --- | --- | --- | --- | --- | --- |
| TyG index | 8.64 | 0.704 | 0.590 | 0.70 | (0.67 – 0.73) | <0.001 |

With age, sex, heart failure, substance use, physical activity, BMI, waist circumferences, SBP, DBP, LDL-C, HDL-C, HbA1c, and eGFR adjustment.

**Supplemental Table 2** The Associations of Each Unit Increase in the TyG Index With Cardiac Variables.

|  | LV mass index | | |  | TDI-e’ | | |  | E/e’ ratio | | |  | LV-GLS | | |
| --- | --- | --- | --- | --- | --- | --- | --- | --- | --- | --- | --- | --- | --- | --- | --- |
|  | β (95% CI) | *P* | *P* for interaction |  | β (95% CI) | *P* | *P* for interaction |  | β (95% CI) | *P* | *P* for interaction |  | β (95% CI) | *P* | *P* for interaction |
| Total | -1.32 (-2.06 – 0.58) | <0.001 |  |  | -0.54 (-0.64 – 0.44) | <0.001 |  |  | 0.21 (0.08 – 0.33) | 0.001 |  |  | 0.38 (0.28 – 0.48) | <0.001 |  |
| HF |  |  |  |  |  |  |  |  |  |  |  |  |  |  |  |
| Stage A | -1.02 (-1.92 - -0.12) | 0.02 | 0.065 |  | -0.58 (-0.72 - -0.45) | <0.001 | 0.68 |  | 0.21 (0.07 – 0.35) | 0.003 | 0.49 |  | 0.39 (0.26 – 0.53) | <0.001 | 0.36 |
| Stage B | -3.73 (-5.86 - -1.61) | 0.001 |  |  | -0.46 (-0.69 - -0.22) | <0.001 |  |  | 0.09 (-0.32 – 0.51) | 0.66 |  |  | 0.42 (0.17 – 0.68) | 0.001 |  |
| CKM syndrome |  |  |  |  |  |  |  |  |  |  |  |  |  |  |  |
| Without | -5.49 (-8.62 - -2.37) | 0.001 | 0.19 |  | -0.47 (-0.94 - -0.01) | 0.04 | 0.69 |  | -0.17 (-0.81 – 0.46) | 0.58 | 0.11 |  | 0.20 (-0.28 – 0.69) | 0.41 | 0.02 |
| With | -1.39 (-2.31 - -0.48) | 0.003 |  |  | -0.53 (-0.65 - -0.41) | <0.001 |  |  | 0.19 (0.03 – 0.35) | 0.01 |  |  | 0.41 (0.28 – 0.53) | <0.001 |  |

Data are presented as β value and 95% confidence intervals (CI) using multiple linear regression analysis with age, sex, heart failure, substance use, physical activity, BMI, waist circumferences, SBP, DBP, LDL-C, HDL-C, HbA1c, and eGFR adjustment.

HF = heart failure; CKM = cardiovascular-kidney-metabolic; LV = left ventricular; TDI = tissue Doppler image; LV-GLS = left ventricle function global longitudinal strain.
